# A tract-specific approach to assessing white matter in preterm infants

**DOI:** 10.1016/j.neuroimage.2017.04.057

**Published:** 2017-08-15

**Authors:** Diliana Pecheva, Paul Yushkevich, Dafnis Batalle, Emer Hughes, Paul Aljabar, Julia Wurie, Joseph V. Hajnal, A. David Edwards, Daniel C. Alexander, Serena J. Counsell, Hui Zhang

**Affiliations:** a Centre for the Developing Brain, Division of Imaging Sciences & Biomedical Engineering, King's College London, UK; b Department of Computer Science and Centre for Medical Image Computing, University College London, UK; c Penn Image Computing and Science Laboratory (PISCL), Department of Radiology, University of Pennsylvania, Philadelphia, USA

**Keywords:** Diffusion weighted MRI, Preterm, Infant, White matter, Tract-specific analysis

## Abstract

Diffusion-weighted imaging (DWI) is becoming an increasingly important tool for studying brain development. DWI analyses relying on manually-drawn regions of interest and tractography using manually-placed waypoints are considered to provide the most accurate characterisation of the underlying brain structure. However, these methods are labour-intensive and become impractical for studies with large cohorts and numerous white matter (WM) tracts. Tract-specific analysis (TSA) is an alternative WM analysis method applicable to large-scale studies that offers potential benefits. TSA produces a skeleton representation of WM tracts and projects the group's diffusion data onto the skeleton for statistical analysis. In this work we evaluate the performance of TSA in analysing preterm infant data against results obtained from native space tractography and tract-based spatial statistics. We evaluate TSA's registration accuracy of WM tracts and assess the agreement between native space data and template space data projected onto WM skeletons, in 12 tracts across 48 preterm neonates. We show that TSA registration provides better WM tract alignment than a previous protocol optimised for neonatal spatial normalisation, and that TSA projects FA values that match well with values derived from native space tractography. We apply TSA for the first time to a preterm neonatal population to study the effects of age at scan on WM tracts around term equivalent age. We demonstrate the effects of age at scan on DTI metrics in commissural, projection and association fibres. We demonstrate the potential of TSA for WM analysis and its suitability for infant studies involving multiple tracts.

## Highlights

• Evaluation of tract-specific analysis (TSA) for white matter studies in infants.
• TSA improves white matter tract alignment over scalar-based registration.
• TSA closely approximates native space tractography DTI values.
• The first application of TSA to a neonatal population.

## Graphical abstract

**Figure:**
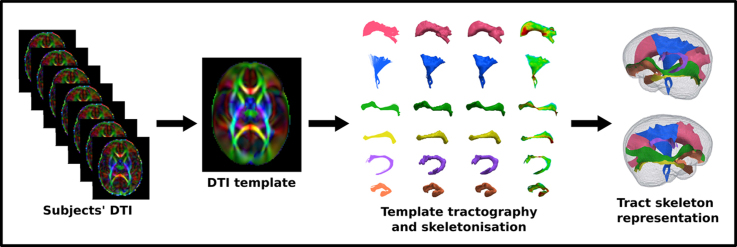

## Introduction

Diffusion-weighted magnetic resonance imaging (dMRI) is increasingly being used to study brain development and injury in infants. Using metrics derived from diffusion tensor imaging (DTI) (Basser et al., 1994) we have gained valuable insights into the effects of maturation and injury on white matter (WM) in healthy and patient infant populations. DTI analyses of WM have been used to assess quantitatively microstructural changes during normal development in infancy (Dubois et al., 2006, Gao et al., 2009) and through childhood to adulthood (Lebel et al., 2008); provide in vivo quantification of the spatio-temporal pattern of WM maturation (Dubois et al., 2008); assess differences in cerebral WM between term and preterm infants (Anjari et al., 2007, Huppi et al., 1998, Rose et al., 2008); and correlate DTI metrics with early developmental outcome in preterm infants (Counsell et al., 2008, van Kooij et al., 2012).

A number of approaches have been used to analyse DTI data during development. Manually-drawn regions of interest (ROI) (Gao et al., 2009, Huppi et al., 1998) or tractography using manually-placed waypoints (Bassi et al., 2008, Dubois et al., 2008, Dubois et al., 2006) are generally assumed to produce anatomically accurate results but these methods become prohibitively labour-intensive for large cohort studies. Subsequently a number of methods have been developed for automatic segmentation of WM tracts (Suarez et al., 2012, Zhang et al., 2010b). However, establishing correspondence between subjects’ WM tracts can be problematic due to inter-subject variability in anatomy and DTI characteristics, which can result in differences in tractography or segmentation. It is possible to average the DTI metrics over the entire tract (Lebel et al., 2008) but localised differences may be missed. Correspondence can be achieved by sampling at equivalent levels along tracts (Groeschel et al., 2014, Verde et al., 2014) or parameterising WM tracts by arc length, essentially reducing entire tracts to a single, core line (Corouge et al., 2006, Goodlett et al., 2009, O'Donnell et al., 2009, Verde et al., 2014, Yeatman et al., 2012). These methods have been used to study neurodevelopment in toddlers (Geng et al., 2012, Goodlett et al., 2009), WM heritability in twin neonates (Lee et al., 2015), infantile Krabbe disease (Gupta et al., 2015), and prenatal exposure to selective serotonin reuptake inhibitors (Jha et al., 2016). However these methods are more suitable for tubular rather than sheet-like tracts. Collapsing tracts such as the corticospinal tract into a single line, especially in the region of the fanning cortical projections, fails to appropriately represent the tract macrostructure and averaging over such a large area may obscure microstructural changes. Moreover, bundles such as the corpus callosum have to be separated into tubular regions and cannot be analysed as a whole.

Exploiting the sheet-like structure of many WM tracts, tract-based spatial statistics (TBSS) was introduced (Smith et al., 2006) and initiated the practice of projecting volumetric data onto a WM skeleton. Although it has proven to be a valuable analysis tool for studying development (Anjari et al., 2007, Ball et al., 2010, Counsell et al., 2008, Rose et al., 2008, van Kooij et al., 2012), recent studies have discussed the potential pitfalls of TBSS (Bach et al., 2014, de Groot et al., 2013, Edden and Jones, 2011, Schwarz et al., 2014, Van Hecke et al., 2010, Zalesky, 2011). A particular limitation of TBSS is a lack of anatomical specificity due to the construction of the skeleton for the entire WM, rather than separately for each individual WM tract. Although TBSS is useful when there is no a priori hypothesis regarding the anatomical location of an effect of interest, it makes it impossible to distinguish between adjacent WM tracts such as the inferior longitudinal and inferior-fronto-occipital fasciculi.

Tract-specific analysis (TSA) (Yushkevich et al., 2008) is an alternative WM analysis method that creates skeleton models of individual WM tracts onto which diffusion data can be projected for statistical analysis. In TSA, subjects are registered to a study-specific template using a tensor-based algorithm (Zhang et al., 2006). Following registration, tracts of interest are delineated from the template using deterministic tractography and manually-drawn regions of interest. From the tractography results, a medial surface is determined for each tract that simultaneously defines its skeleton and boundary (Yushkevich and Zhang, 2013). The skeleton also describes local tract thickness via the radius function defined as equal to the radius of the maximal inscribed sphere within the boundary centred at that point on the skeleton. Diffusion data from every subject is then projected onto the skeleton, similarly to TBSS. TSA samples data to be projected onto each point of the skeleton by searching along the unit normal from that point to the tract boundary. The tract boundary defines the stopping criteria. This aims to limit potential voxel misassignment from neighbouring tracts. The TSA framework allows for either a maximum-value or mean-value data projection strategy. In the maximum-value strategy, the tensor with the highest FA value is selected. In the mean-value strategy, the average tensor is computed and from this average tensor, scalars such as FA are computed. Statistical analysis of projected diffusion data is then carried out at each point on the skeleton. The key aspects of the TSA and TBSS pipelines and their differences are summarised in Table 1.

**Table 1:** A summary of the key aspects of the TSA and TBSS pipelines.

**Aspect**	**TSA**	**TBSS**
Registration	Tensor-based	Scalar-based (FA)
Search direction	Perpendicular to the skeleton surface	Direction of maximum change within a local 3x3×3 voxel neighbourhood.
Choice of voxel to project	Maximum FA tensor or mean tensor	Maximum FA tensor
Stopping criteria	Tract boundary	Skeleton distance map
Statistical resolution	Point on surface	Voxel
Multiple comparisons	Suprathreshold cluster analysis	Threshold-free cluster enhancement

TSA offers potential advantages as an analysis tool. It is automated therefore reducing the time cost and inter-rater variability which affect manual-input methods. It characterises WM tracts as surfaces rather than aggregating tracts into a single core line thereby capturing the overall tract morphology. Theoretically TSA also offers improvements over TBSS by (i) employing a tensor-based rather than scalar-based registration; (ii) defining tracts individually and so making it possible to distinguish between adjacent tracts; and (iii) having a data projection search stopping criteria intended to limit crossing over into neighbouring tracts. TSA has been successfully applied to study pathologies such as paediatric chromosome 22q11.2 deletion syndrome (Yushkevich et al., 2008) and amyotrophic lateral sclerosis (Zhang et al., 2010a), and changes in DTI metrics over the lifespan (Chen et al., 2016), however has not been previously applied to study infant populations. Moreover, the performance of TSA has not been assessed extensively.

The aim of this study is to evaluate the performance of TSA within the context of preterm infant data. We compare TSA with native space tractography as a gold standard, and with TBSS, a similar and widely-used method. Despite some known limitations, TBSS remains a widely-used tool, having been cited over 3000 times (618 alone since 2016). Our evaluation of TSA involves (i) an assessment of TSA's ability to align WM tracts from different subjects and the accuracy of its data projection step in comparison to TBSS; and (ii) an application of TSA for the first time to a cohort of preterm infants at term equivalent age to determine whether TSA is able to detect developmental changes in diffusion properties of WM tracts.

## Methods

### Subjects

Permission for this study was granted by Queen Charlotte's and Hammersmith Hospitals Research Ethics Committee (07/H0704/99) and written parental consent was acquired prior to imaging. MR data were collected from 53 preterm subjects who were imaged between February and July 2013. All images were reviewed by an experienced perinatal neuroradiologist and cases with major focal lesions were excluded. Five data-sets were excluded; 2 unilateral haemorrhagic infarction, 1 cerebellar infarct, 1 cerebellar haemorrhage and 1 infant had temporal and cerebellar haemorrhages with cerebellar hypertrophy. 48 subjects (23 female) born at a median (range) gestational age (GA) of 30.6 (24.0–32.9) weeks and imaged at a median age of 41.9 (38.6–47.1 weeks) weeks post-menstrual age (PMA) were analysed in this study. The perinatal characteristics of the study group are summarised in Table 2.

**Table 2:** Perinatal characteristics of the study group.

**Perinatal clinical characteristic**	
Median (range) gestational age at birth	30.64 (24–32.86) weeks
Median (range) postmenstrual age at scan	41.93 (38.57 – 47.14) weeks
Median (range) day age at scan	84 (142 – 48) days
Median (range) birthweight	1218 (655–1960) grams
Median (range) days of ventilation	0 (0 – 40) days
Small for gestational age^a^ (number of infants)	13

a Defined as <10th birthweight percentile.

### Data acquisition

MR imaging was performed on a 3-T MR system sited on the neonatal intensive care unit. T1- and T2-weighted MR imaging and single shot echo planar dMRI data were acquired using an 8-channel phased array head coil. The pulse sequence parameters were as follows. 3D MPRAGE: repetition time (TR) = 17 ms, echo time (TE) = 4.6 ms, flip angle $13^{\circ }$, voxel size : 0.82×0.82×0.8 mm T2 weighted fast-spin echo imaging: TR = 8670 ms, TE = 160 ms, flip angle $90^{\circ }$, slice thickness 2 mm with 1 mm overlapping slices, in-plane resolution 1.14 × 1.14 mm. dMRI was acquired in the transverse plane in 32 non-collinear directions using the following parameters: TR = 8000 ms, TE = 49 ms, voxel size: 2 mm isotropic, b-value: 750 s/mm^2^, SENSE factor of 2.

All examinations were supervised by a paediatrician experienced in MR imaging procedures. Infants were sedated with oral chloral hydrate (25–50 mg/kg) prior to scanning and pulse oximetry, temperature, and electrocardiography data were monitored throughout. Ear protection was used, comprising earplugs moulded from a silicone-based putty (President Putty, Coltene Whaledent, Mahwah, NJ, USA) placed in the external auditory meatus and neonatal earmuffs (MiniMuffs, Natus Medical Inc., San Carlos, CA, USA).

### Diffusion weighted image processing

Diffusion-weighted images were visually inspected in 3 orthogonal planes for the presence of motion artefact and corrupt diffusion weighted volumes were excluded before tensor fitting. 33 subjects had no volumes excluded, eight subjects had one volume excluded, three subjects had two volumes excluded, three subjects had three volumes excluded and one subject had four volumes excluded. Non-brain tissue was removed using BET (Smith, 2002), images were corrected for eddy current artefacts using *eddy* (Andersson and Sotiropoulos, 2015) and the tensor model was fitted using *dtifit* from FSL (FMRIB, Oxford, http://fsl.fmrib.ox.ac.uk). Signal-to-noise-ratio (SNR) was calculated for each subject from the raw DW data. A 5×5 voxel ROI was manually placed in the central corona radiata of the b=0 volume and SNR was calculated as the mean signal divided by the standard deviation. For each subject deterministic tractography based on the FACT approach (Mori et al., 1999) (part of DTI-TK http://dti-tk.sf.net) was used to delineate WM tracts in native space. Each subject's FA map was thresholded at 0.1 and whole brain tractography was seeded from each voxel with tracking parameters: maximum angle threshold of 45 and minimum FA threshold of 0.1. ROIs used to delineate tracts in native space were drawn manually for each subject, according to the protocol outlined in Wakana et al. (2007). Separate ROIs were drawn for TSA. Placement of the ROIs is described in Table 3. The tracts delineated were the bilateral corticospinal tract (CST), inferior fronto-occipital fasciculus (IFOF), inferior longitudinal fasciculus (ILF), superior longitudinal fasciculus (SLF), uncinate fasciculus (UNC) and genu and splenium of the corpus callosum (CC), to include commissural, projection and association tracts in our comparison.

**Table 3:** ROI placement for tractography.

**Tract**	**First ROI**	**Second ROI**	**Exclusion ROI**
CC genu	The CC is identified in the mid-sagittal plane and only the genu is selected	N/A	Exclude fibres that project posteriorly along the fornix.
CC splenium	The CC is identified in the mid-sagittal plane and only the splenium is selected.	N/A	Exclude fibres that project inferiorly along association fibres.
CC (whole)	The CC is identified in the mid-sagittal plane.	N/A	Exclude fibres passing through the cingulum and fornix.
CST	CST is identified in the axial plane at the level of the of the decussation of the superior cerebellar peduncle.	Projections to the cortex are identified in the axial plane at the level of the central semiovale.	Exclude fibres crossing into the opposite hemisphere and into the cerebellum.
IFOF	The occipital lobe is selected in the coronal plane identified halfway between the posterior edge of the cingulum and the posterior of the brain.	The entire hemisphere in the coronal plane at the level of the genu of the CC identified in the mid-sagittal slice.	Exclude fibres crossing medially through the anterior commissure.
ILF	The entire hemisphere is selected in the coronal plane at the posterior edge of the cingulum identified at the mid-sagittal slice.	The entire temporal lobe identified in the coronal plane at the level where the frontal and temporal lobe are no longer connected.	Exclude fibres that track medially into the fornix and CC.
SLF	The SLF is identified in the coronal plane at the lowest axial level in which the fornix can be identified as a single structure.	Projections that pass through the coronal plane at the level of the splenium of the CC identified in the mid-sagittal slice.	Exclude fibers that project into the external capsule.
UNC	The entire temporal lobe identified in the coronal plane at the level where the frontal and temporal lobe are no longer connected.	All the projections into the frontal lobe.	Exclude fibres which project into the anterior limb of the external capsule and posteriorly.

### Evaluation of TSA

To evaluate TSA, we assessed two key aspects of the pipeline that are comparable in both TSA and TBSS. To determine how well TSA is able to align subjects’ WM tracts we compared the standard TSA registration with an existing protocol that was optimised for neonatal spatial normalisation (Ball et al., 2010). To determine how accurately the TSA skeletons represent subjects’ WM tracts we compared the mean FA values in each tract and the distributions of FA values over the whole tract projected by TSA with those calculated from native space tractography, which we take to be the ground truth.

#### Registration comparison

WM tracts delineated in native space were warped using the transformations from TSA's tensor-based registration, DTI-TK, and the scalar-based registration from an optimised neonatal protocol to their respective template spaces. The scalar-based registration is an adaptation of the FNIRT (FMRIB, Oxford, http://fsl.fmrib.ox.ac.uk) registration used in TBSS (Ball et al., 2010). Each subject's tracts were converted into binary ROIs which were compared using Dice scores. For each of the two registration methods, a Dice score was calculated pairwise between each subject and all other subjects, measuring the degree of overlap between two subjects’ WM tracts at the voxel level. The median Dice score was calculated per tract for each subject. The Wilcoxon signed rank test was used to compare the differences between the Dice scores from the two registrations.

#### Data projection

To determine how accurately TSA projects each subject's diffusion data, we compared projected FA values, in template space following registration, with those calculated from the subjects’ native space tractography. We also compared TSA's data projection step with the data projection step carried out in TBSS, to determine how TSA performs with respect to a similar and widely used method. While previous studies have investigated the accuracy of data projection step in TBSS in terms of alleviating misregistrations (Zalesky, 2011) and voxel misassignment following projection (Bach et al., 2014), none have investigated how template space data projected onto the skeleton representation of WM deviates from native space data for either method. For each subject, we calculated the mean of the FA values as well as the distribution of FA values over a tract derived from native space tractography, and projected by TSA and TBSS. This was done for the left and right CST, IFOF, ILF, UNC and genu and splenium of the CC. We take the FA values calculated from each subjects’ native space tractography as our ground truth. For each subject, and across all eight tracts, we calculated the Bhattacharyya distances between the distribution of values over the tract derived from native space tractography and TSA, and between native space tractography and TBSS. The Wilcoxon signed rank test was used to compare the difference in the resulting paired Bhattacharyya distances. The tract-averaged FA values provide a summary of the differences between the methods, whereas the Bhattacharryya distance calculation assesses the difference between TSA and TBSS's deviation from the native space data over the entire distribution of FA values within a tract.

**Fig. 1:**
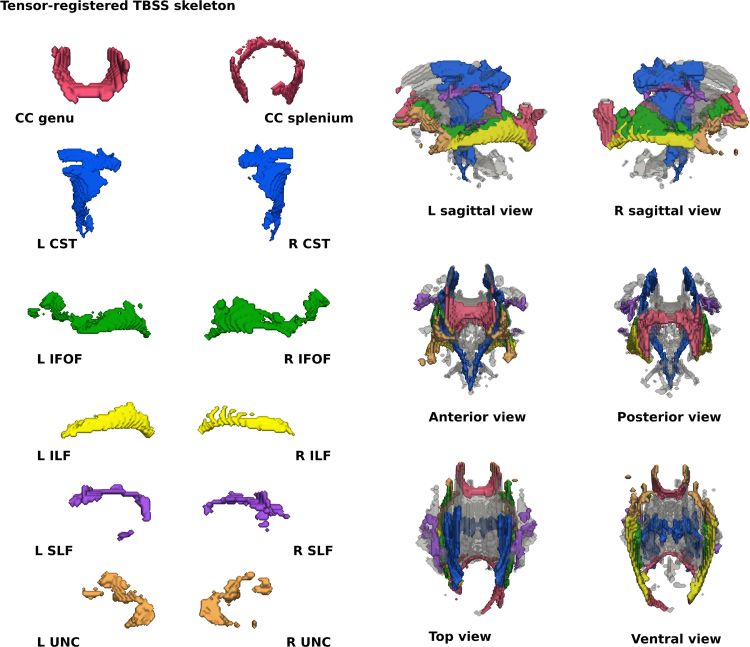
The tract skeletons from TBSS. The top four rows show the tract skeletons evaluated and the bottom three rows show the tracts overlaid on the whole WM skeleton (shown in grey).

Some of the differences in projected values will be due to differences in registration since TBSS uses a different registration to TSA. As we are only interested in the data projection step, TSA's registration was incorporated into the TBSS pipeline. TBSS was also adapted to make the TBSS skeleton “tract specific” to allow a comparison with TSA and native space tractography. The tracts from the deterministic tractography identified in the TSA template were warped to the TBSS mean FA template. A binary segmentation of each tract was then overlapped with the TBSS skeleton. The voxels in the TBSS skeleton that overlap with the binary segmentation of a particular tract were assigned to that tract. This produced a mean FA value and distribution of FA values for the different tracts for each subject. The skeleton segmentation for the TBSS skeleton is shown in Fig. 1.

### Correlation of DT-derived measures and post-menstrual age at scan using TSA

We study the effects of PMA at scan on DTI-derived measures in a number of commissural, association and projection fibres. We expect to observe an increase in FA and a decrease in MD and RD with increasing PMA at scan, consistent with the maturation pattern observed previously. Each infant's diffusion tensor images were registered to a study-specific template that was created as an iteratively-refined average of all subjects’ tensor images (Zhang et al., 2007). Deterministic tractography was carried out in the template to delineate the CC, CST, IFOF, ILF, UNC and SLF using the same protocol as in Section 2.3. The tract skeleton and boundary were derived from the template tractography results for each tract. Each subject's registered DTI data were projected onto the WM skeletons using the maximum-value strategy. Linear regression analysis carried out on the tract skeletons was used to assess the correlation between PMA at scan and FA, axial, radial and mean diffusivities (AD, RD, MD) with GA at birth as a covariate. We corrected for multiple comparisons using non-parametric permutation-based suprathreshold cluster analysis (Nichols and Holmes, 2002) with family-wise error rate (FWER) correction. We also analysed the effects of GA at birth on DTI metrics, with PMA at scan as a covariate, and the effects of post-natal day at scan with GA at birth and PMA at scan as covariates.

## Results

### Evaluation of TSA

#### Registration comparison

**Fig. 2:**
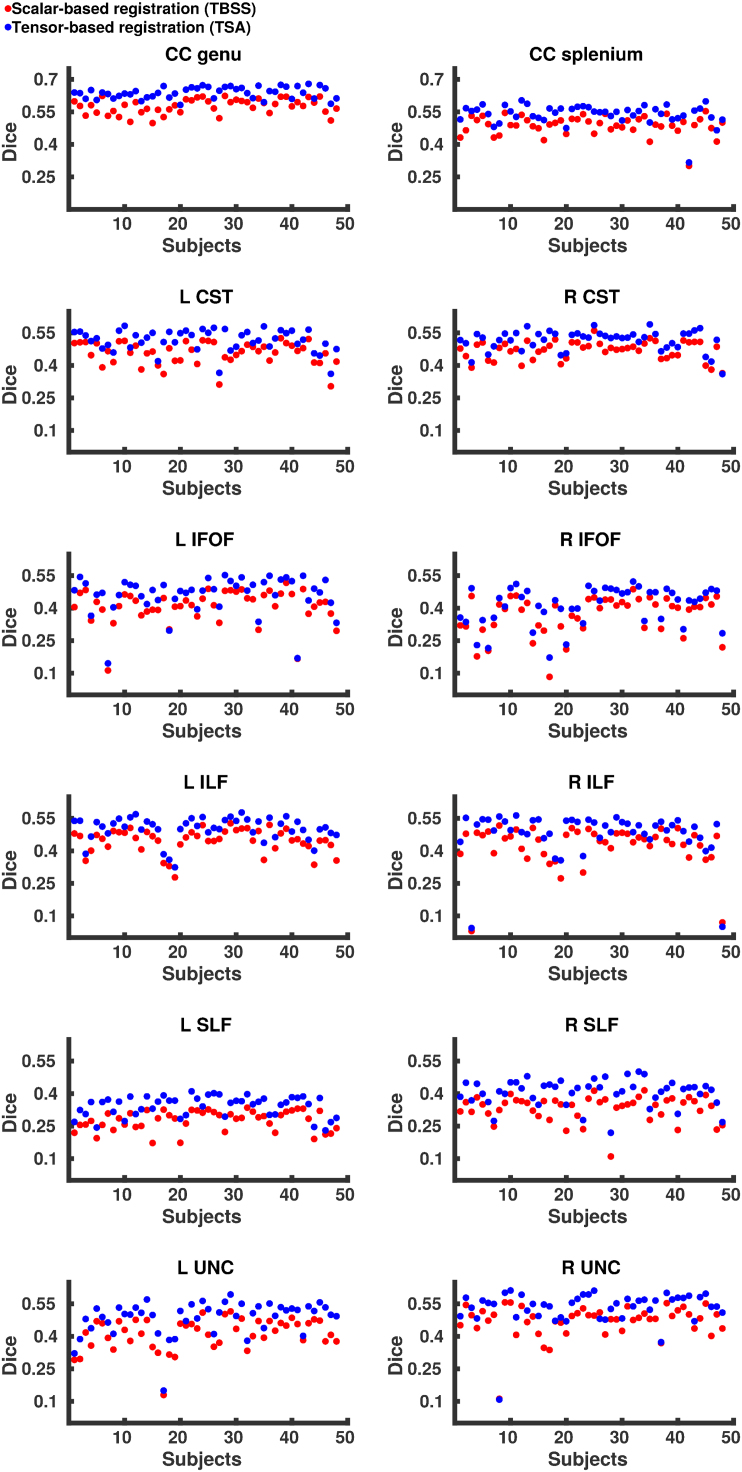
Median Dice scores for each subject over eight tracts following tensor-based and scalar-based registration.

**Fig. 3:**
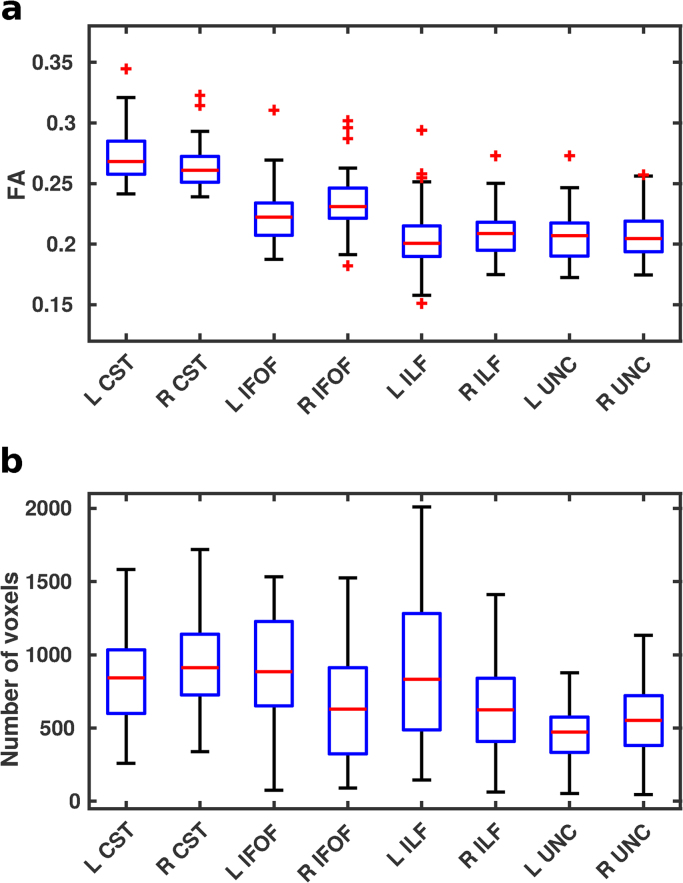
The variability across subjects’ native space tractography for the left and right CST, IFOF, ILF and UNC shown in terms of a. FA values averaged over the entire tract and b. the total number of voxels included.

Fig. 2 shows the median Dice scores following tensor-based and scalar-based registration for the eight tracts, representing the degree of alignment between subjects. For each tract, the Dice scores for the tensor-based registration were higher (*p* < 0.001, summarised in Table 4) than those for the scalar-based registration, demonstrating that TSA's tensor-based registration provides better alignment consistently over all tracts. However, it should be noted that for the subjects with the very lowest Dice scores the two registration techniques have very similar scores. Although native space tractography produced anatomically plausible results for these subjects and in these tracts, they contained fewer voxels resulting in a lower degree of overlap with other subjects’ tracts. Indeed, there was notable variability across subjects within the native space tracts in terms of both FA values and number of voxels (Fig. 3), however SNR was variable across subjects with mean SNR=22.2 (range=11.3 – 48.6).

**Table 4:** p-Values from the Wilcoxon signed rank test comparing Dice scores from the tensor-based and scalar-based registration.

**Tract**	**p-Value**
CC genu	1.63 × 10^−9^
CC splenium	1.63 × 10^−9^
L CST	1.63 × 10^−9^
R CST	1.74 × 10^−9^
L IFOF	1.85 × 10^−9^
R IFOF	1.63 × 10^−9^
L ILF	1.63 × 10^−9^
R ILF	2.10 × 10^−9^
L SLF	1.74 × 10^−9^
R SLF	1.63 × 10^−9^
L UNC	1.63 × 10^−9^
R UNC	2.54 × 10^−9^

#### Data projection

**Fig. 4:**
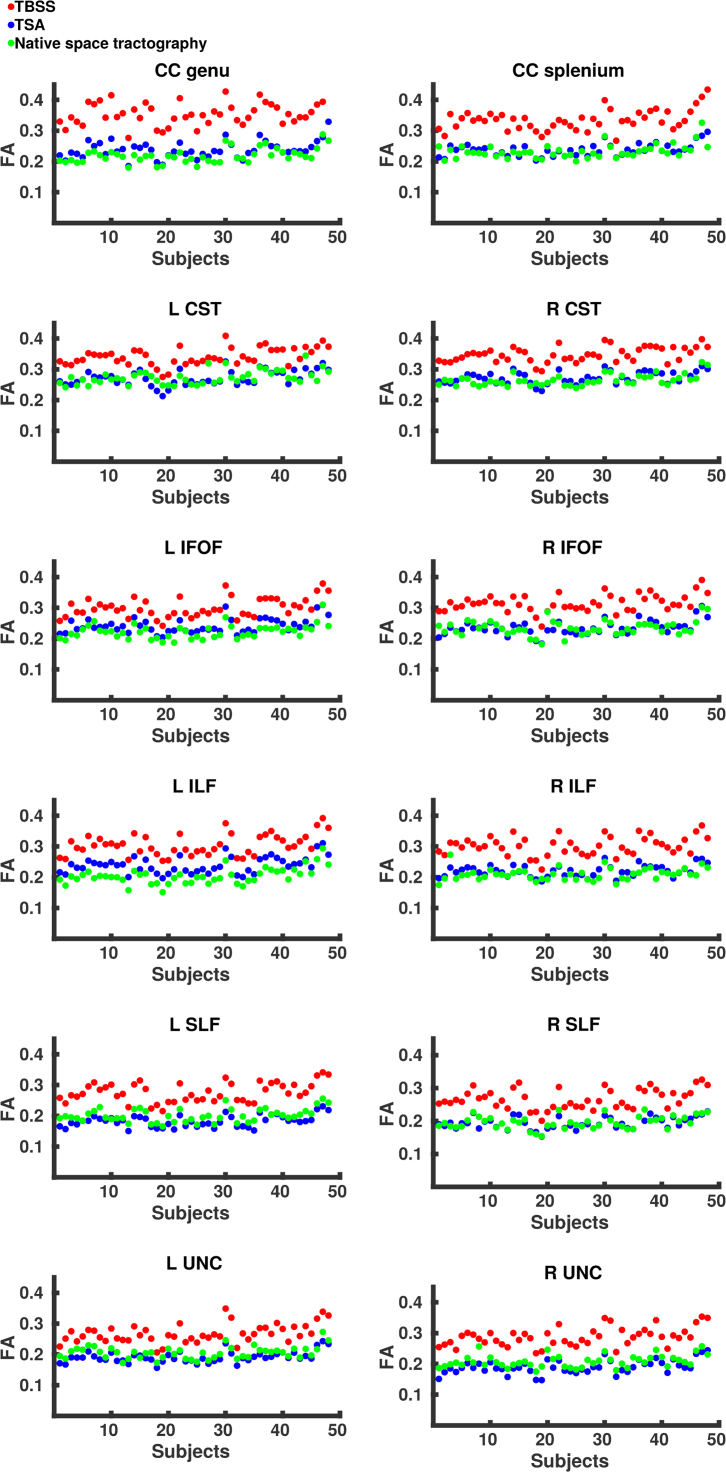
The mean FA values for each subject across eight tracts as estimated by native space tractography (green), TSA (blue) and TBSS (red). (For interpretation of the references to color in this figure legend, the reader is referred to the web version of this article.)

**Fig. 5:**
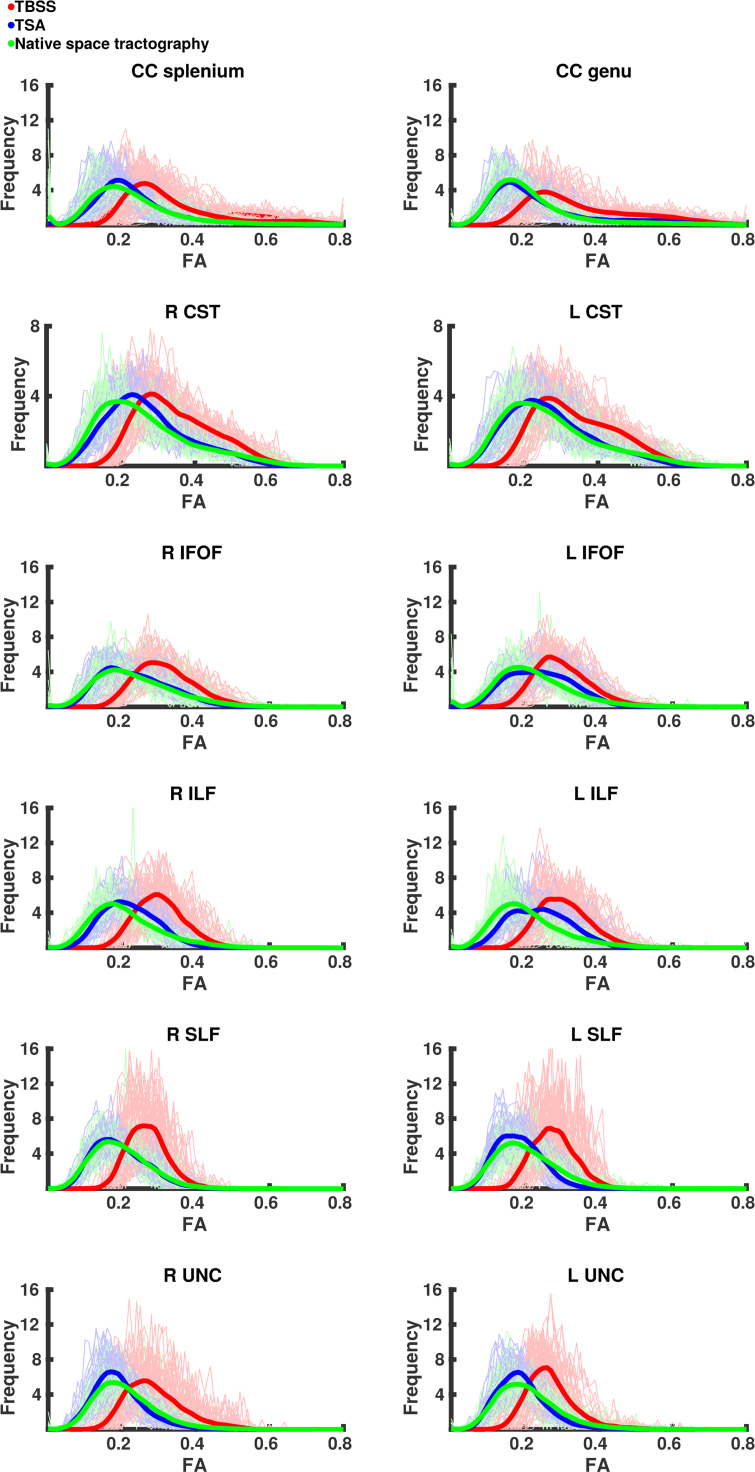
The distribution of FA values for all subjects across eight tracts as estimated by native space tractography (green), TSA (blue) and TBSS (red). Normalised histograms for each subject (semi-transparent green, blue and red) were averaged and smoothed to represent the general trend (green, blue and red lines). (For interpretation of the references to color in this figure legend, the reader is referred to the web version of this article.)

The means and distributions of FA values from different WM tracts are shown in Fig. 4, Fig. 5, respectively. Fig. 4 shows the mean values per subject for each tract as derived from native space tractography and TSA. Overall TSA projects FA values similar to those obtained from the native space tractography, however these were still significantly different from native space-derived FA values in all but three tracts – left CST, right IFOF and right SLF (Table 5). TBSS projects FA values significantly higher than those produced by both native space tractography and TSA.

**Table 5:** p-values from the Wilcoxon signed rank test comparing mean FA values between native space and TBSS, native space and TSA, and TSA and TBSS.

**Tract**	**Native space vs TBSS**	**Native space vs TSA**	**TSA vs TBSS**
	**(p-value)**	**(p-value)**	**(p-value)**
CC genu	1.63 × 10^−9^	9.84 × 10^−9^	1.63 × 10^−9^
CC splenium	1.63 × 10^−9^	0.0058	1.63 × 10^−9^
L CST	1.63 × 10^−9^	0.87	1.63 × 10^−9^
R CST	1.63 × 10^−9^	2.41 × 10^−4^	1.63 × 10^−9^
L IFOF	1.63 × 10^−9^	1.69 × 10^−8^	1.63 × 10^−9^
R IFOF	1.63 × 10^−9^	0.74	1.63 × 10^−9^
L ILF	1.63 × 10^−9^	1.63 × 10^−9^	1.63 × 10^−9^
R ILF	1.63 × 10^−9^	3.22 × 10^−6^	1.63 × 10^−9^
L SLF	1.63 × 10^−9^	2.23 × 10^−9^	1.63 × 10^−9^
R SLF	1.63 × 10^−9^	0.18	1.63 × 10^−9^
L UNC	1.63 × 10^−9^	1.90 × 10^−8^	1.63 × 10^−9^
R UNC	1.63 × 10^−9^	5.69 × 10^−9^	1.63 × 10^−9^

**Fig. 6:**
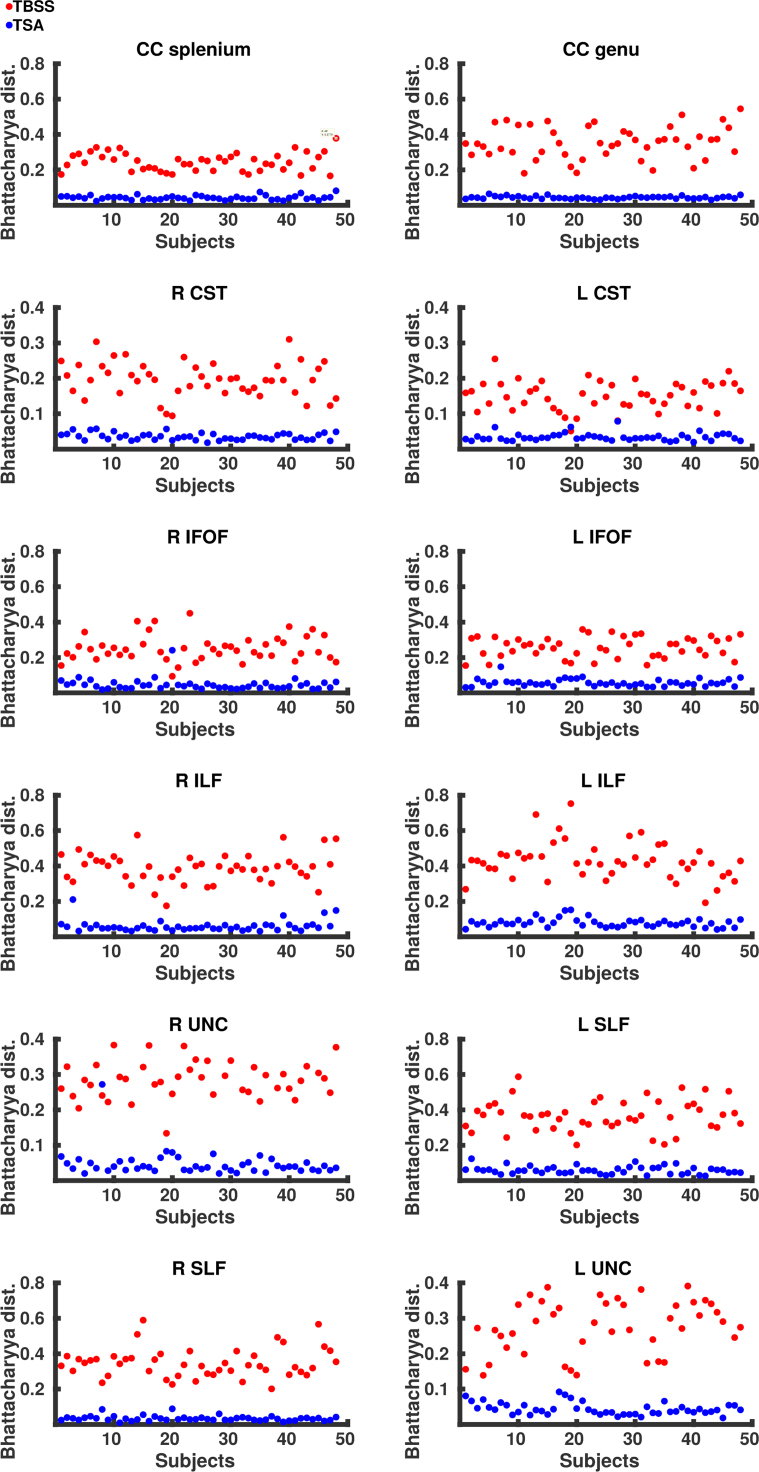
The Bhattacharyya distance between native space tractography and TSA (blue) and TBSS (red). (For interpretation of the references to color in this figure legend, the reader is referred to the web version of this article.)

Fig. 5 shows the normalised histograms for each subject from the native space tractography, TSA and TBSS across eight tracts. Overall the distributions of FA values across the tracts derived from TSA are in close agreement with those obtained from native space. Although the TSA and native space tractography distributions are similar, there are some differences, most noticeably for the left IFOF and ILF where TSA overestimates FA values. The Bhattacharyya distances between native space tractography and TSA, and native space tractography and TBSS are shown for each subject in Fig. 6 and summarised in Table 6. The Bhattacharyya distances between native space tractography and TBSS were significantly greater (p < 0.001), than that between native space tractography and TSA, which were close to zero for every tract.

**Table 6:** Summary of the Bhattacharyya distances.

**Tract**		**TSA**	**TBSS**	**p-value**
CC genu	Mean	0.044	0.354	1.63 × 10^−9^
	SD	0.008	0.091	
				
CC splenium	Mean	0.043	0.244	1.63 × 10^−9^
	SD	0.012	0.051	
				
L CST	Mean	0.031	0.151	1.85 × 10^−9^
	SD	0.011	0.041	
				
R CST	Mean	0.036	0.197	1.63 × 10^−9^
	SD	0.01	0.049	
				
L ILF	Mean	0.079	0.429	1.63 × 10^−9^
	SD	0.025	0.106	
				
R ILF	Mean	0.06	0.388	1.63 × 10^−9^
	SD	0.033	0.083	
				
L IFOF	Mean	0.057	0.258	1.63 × 10^−9^
	SD	0.021	0.058	
				
R IFOF	Mean	0.047	0.253	2.88 × 10^−9^
	SD	0.034	0.073	
				
L SLF	Mean	0.059	0.368	1.63 × 10^−9^
	SD	0.022	0.087	
				
R SLF	Mean	0.034	0.348	1.63 × 10^−9^
	SD	0.015	0.083	
				
L UNC	Mean	0.044	0.290	1.63 × 10^−9^
	SD	0.041	0.086	
				
R UNC	Mean	0.047	0.299	1.74 × 10^−9^
	SD	0.037	0.065	

### Correlation of DT-derived measures and age at scan using TSA

**Fig. 7:**
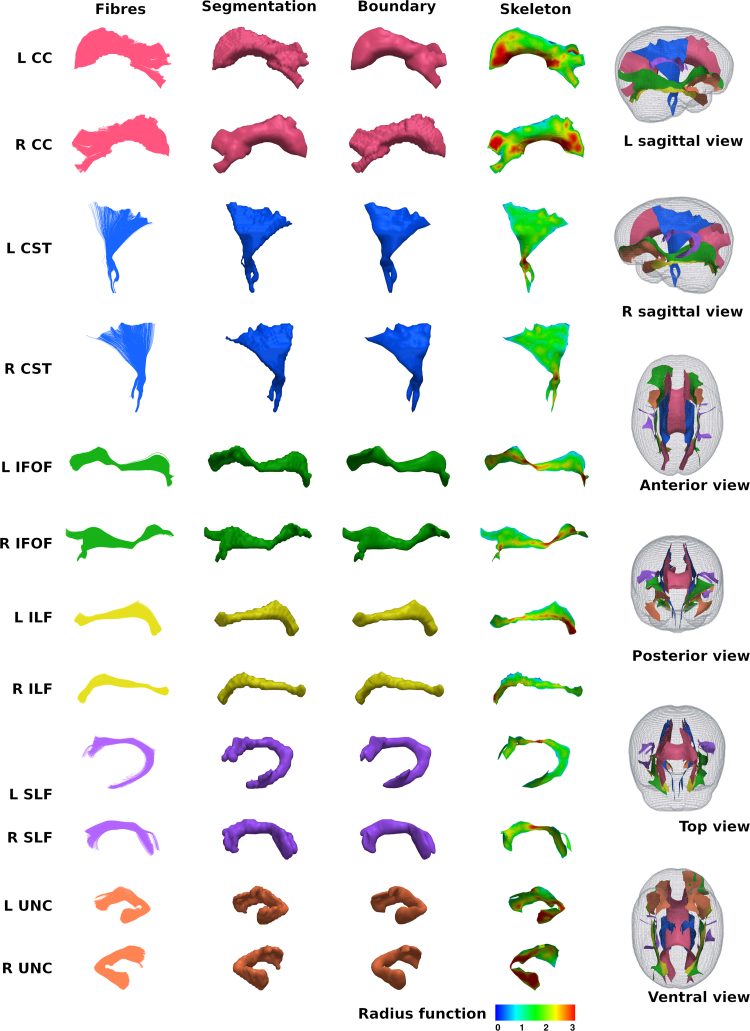
The TSA model fitting results for the left and right CC, CST, IFO, ILF, SLF and UNC.

**Fig. 8:**
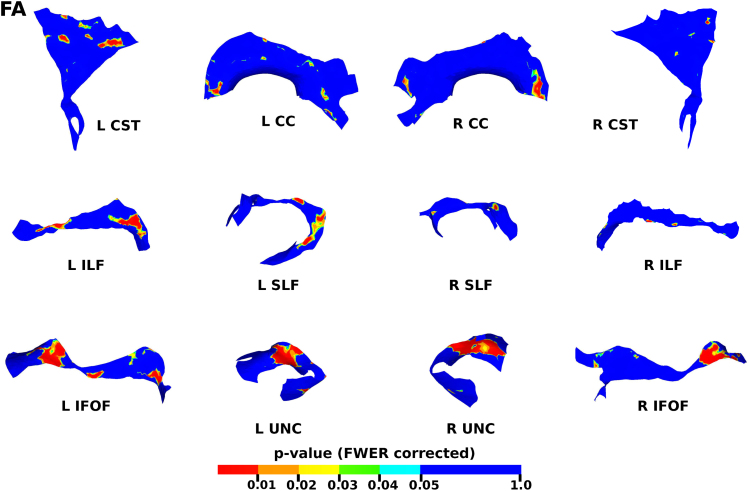
The results of the cluster analysis correlating PMA with FA at each point within the tracts. Statistically significant regions are shown in red (p≤0.01), orange (0.01<p≤0.02), yellow (0.02<p≤0.03), green (0.03<p≤0.04) and light blue (0.04<p≤0.05). (For interpretation of the references to color in this figure legend, the reader is referred to the web version of this article.)

**Fig. 9:**
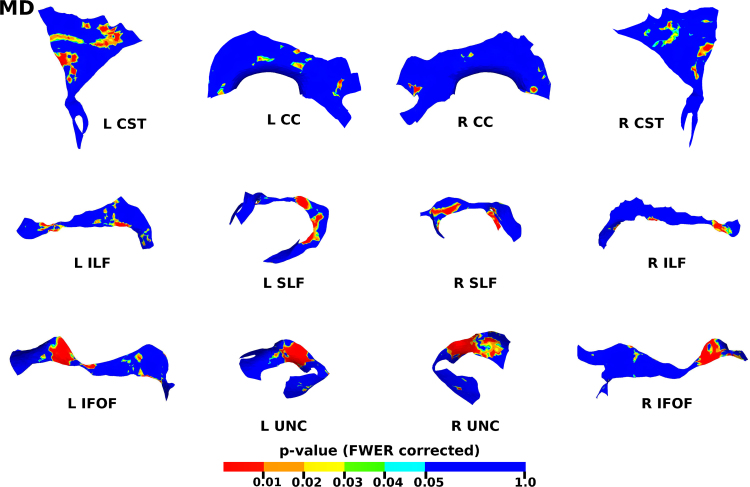
The results of the cluster analysis correlating PMA with MD at each point within the tracts. Statistically significant regions are shown in red (p≤0.01), orange (0.01<p≤0.02), yellow (0.02<p≤0.03), green (0.03<p≤0.04) and light blue (0.04<p≤0.05). (For interpretation of the references to color in this figure legend, the reader is referred to the web version of this article.)

**Fig. 10:**
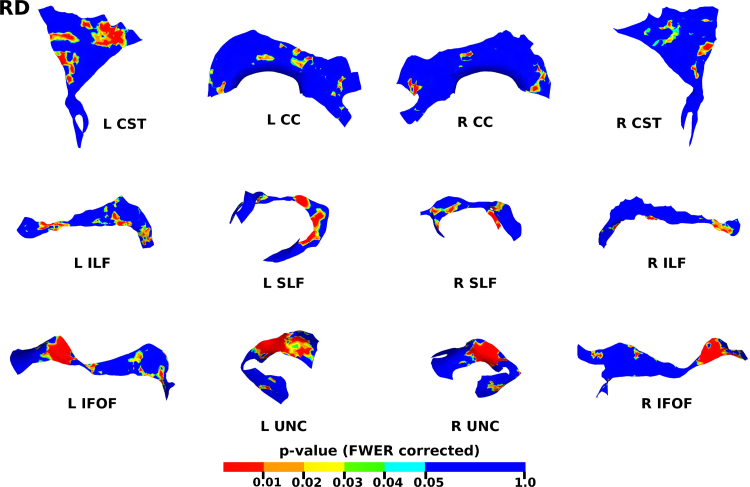
The results of the cluster analysis correlating PMA with RD at each point within the tracts. Statistically significant regions are shown in red (p≤0.01), orange (0.01<p≤0.02), yellow (0.02<p≤0.03), green (0.03<p≤0.04) and light blue (0.04<p≤0.05). (For interpretation of the references to color in this figure legend, the reader is referred to the web version of this article.)

**Fig. 11:**
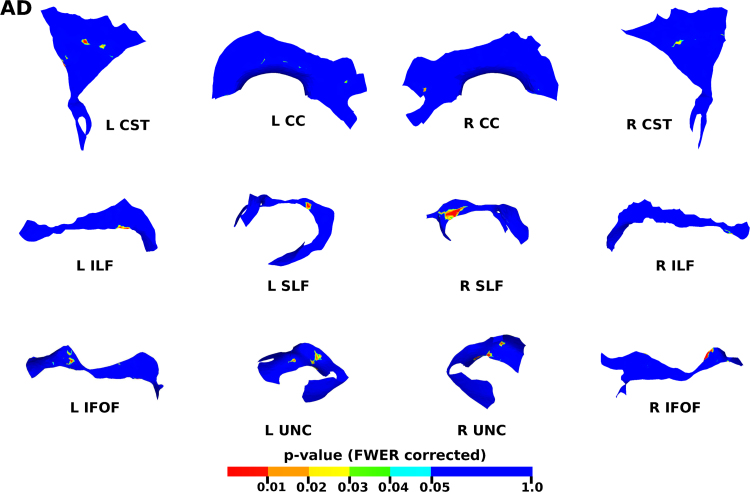
The results of the cluster analysis correlating PMA with AD at each point within the tracts. Statistically significant regions are shown in red (p≤0.01), orange (0.01<p≤0.02), yellow (0.02<p≤0.03), green (0.03<p≤0.04) and light blue (0.04<p≤0.05). (For interpretation of the references to color in this figure legend, the reader is referred to the web version of this article.)

**Fig. 12:**
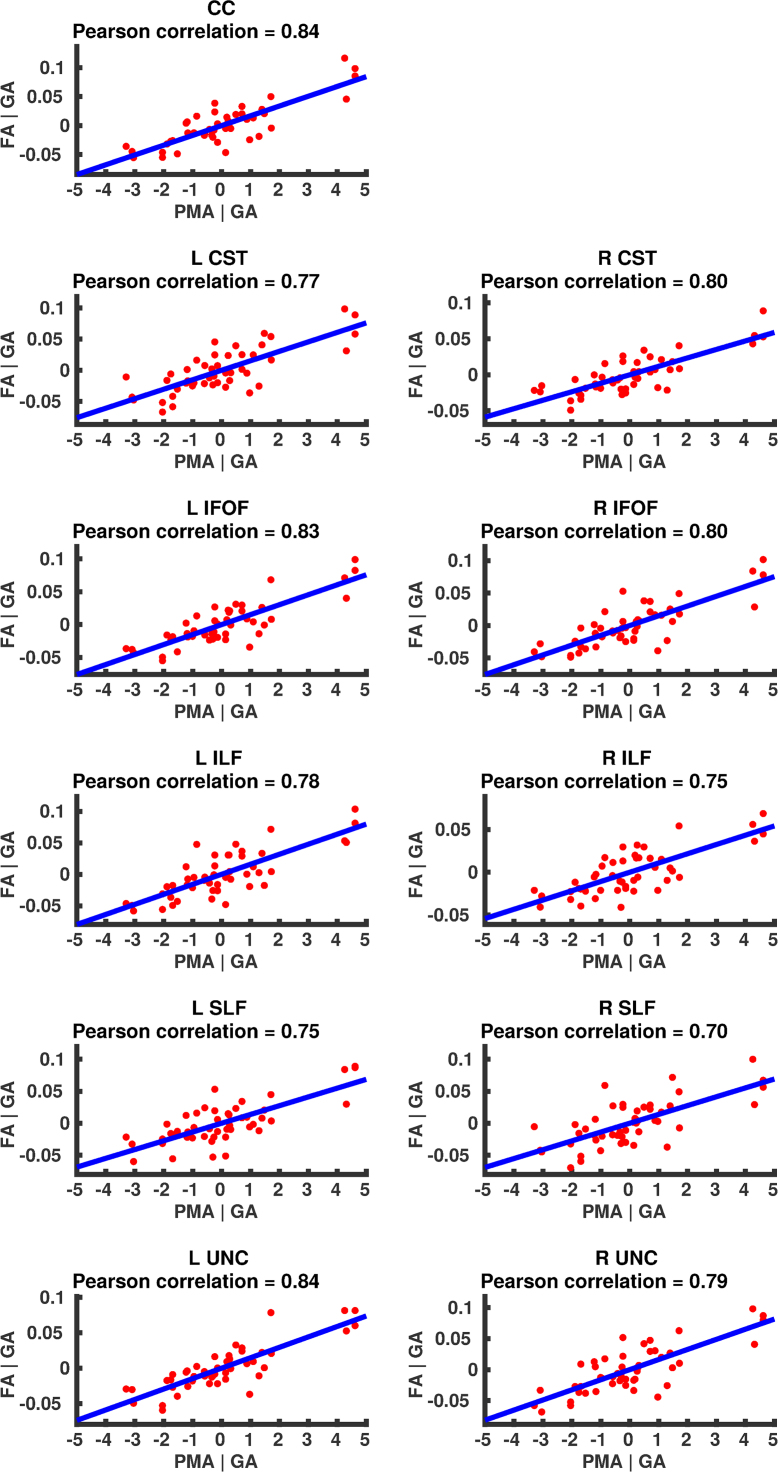
Partial regression plots showing the linear relationship between FA and PMA from the regions showing significant correlation for the CC, CST, IFOF, ILF, SLF and UNC.

**Fig. 13:**
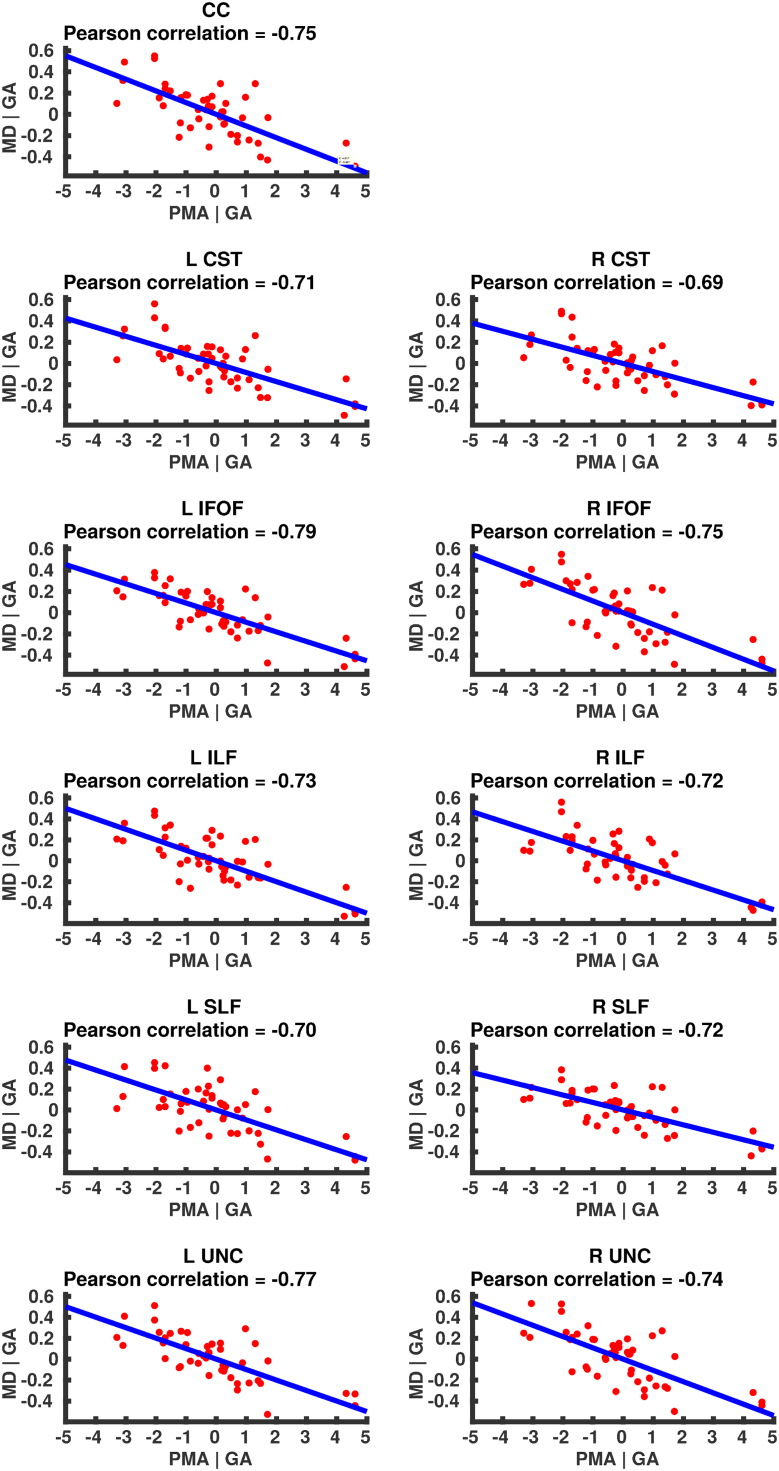
Partial regression plots showing the linear relationship between MD and PMA from the regions showing significant correlation for the CC, CST, IFOF, ILF, SLF and UNC.

**Fig. 14:**
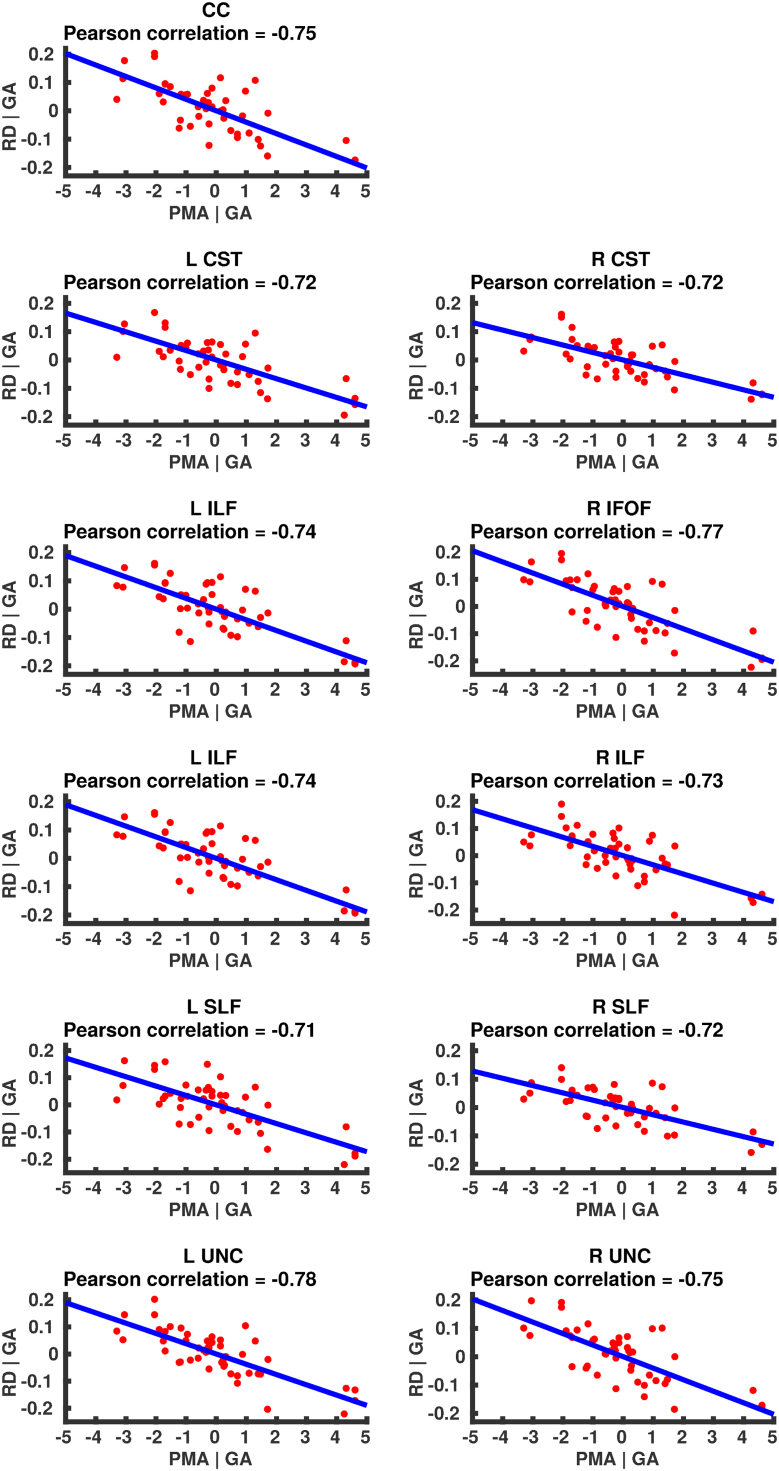
Partial regression plots showing the linear relationship between RD and PMA from the regions showing significant correlation for the CC, CST, IFOF, ILF, SLF and UNC.

**Fig. 15:**
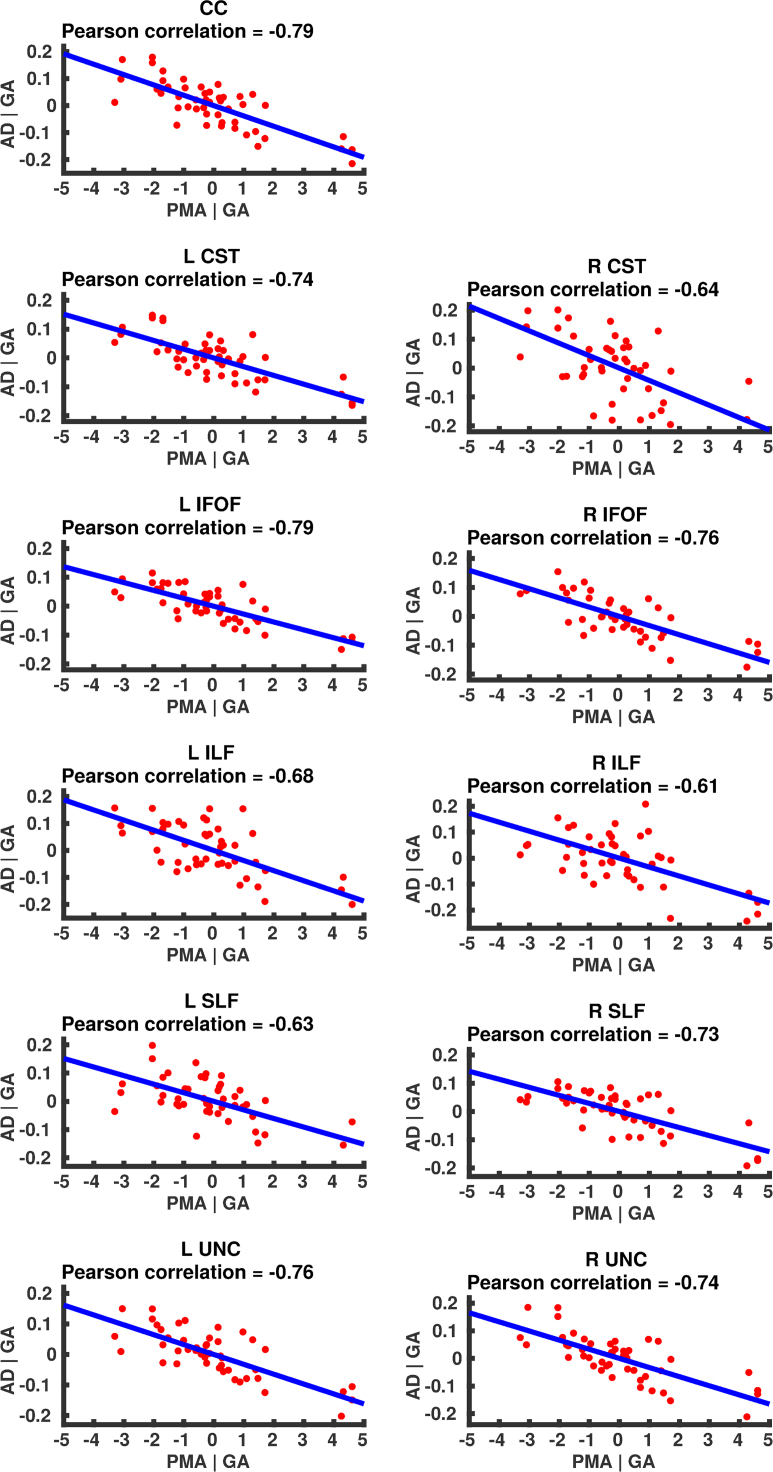
Partial regression plots showing the linear relationship between AD and PMA from the regions showing significant correlation for the CC, CST, IFOF, ILF, SLF and UNC.

The results from the TSA model fitting are shown in Fig. 7. All tracts were wholly reconstructed apart from the trajectories into the temporal lobe for the right SLF. The results from the TSA statistical analysis of DTI metrics are shown in Fig. 8, Fig. 9, Fig. 10, Fig. 11, Fig. 12, Fig. 13, Fig. 14, Fig. 15, showing clusters where DTI metrics were significantly correlated with PMA at scan, FWER-corrected *p*=0.05. TSA shows increases in FA, decreases in MD and RD with increasing PMA at scan, and limited negative correlation with AD. There was no negative correlations between FA and PMA and no positive correlations between AD, MD or RD and PMA. There was no significant correlation between GA at birth and DTI metrics, and there was no significant correlation between postnatal day at scan and DTI metrics.

## Discussion

### Evaluation of TSA

We have evaluated the performance of TSA in analysing preterm infant dMRI data in comparison to TBSS, with native space tractography as our gold standard. The registration comparison shows that TSA's tensor-based registration improves WM tract alignment over TBSS's scalar-based registration. The results from the data projection step show that TSA can approximate native space tractography FA values more closely than TBSS. We applied TSA to the preterm infant population for the first time to study the association between PMA at scan DTI metrics. The results show decreases in RD, MD and AD, and an increase in FA with increasing PMA.

#### Registration comparison

The improvements in alignment seen with TSA's registration are likely to be due to both leveraging the full tensor information and how the template is constructed. Scalar-based registration algorithms discard orientation information, making it difficult to distinguish neighbouring tracts with similar FA values but different orientations, and utilising orientation information during registration significantly improves alignment (Van Hecke et al., 2007). Tensor-based algorithms use the full tensor features resulting in better alignment of the dominant diffusion orientation (Zhang et al., 2007) which may help distinguish neighbouring tracts (Bach et al. 2014). The tensor-based algorithm from TSA has previously been shown to improve registration in adults (Keihaninejad et al., 2013). However infant population registrations require further consideration because the lower contrast and resolution in neonatal scans can be problematic for registration (Ball et al., 2010). Wang et al. (2011) showed that the registration used in TSA outperformed other registration algorithms, including FNRIT, in neonates with Krabbe disease. Their analysis included 10 subjects, was limited to 4 WM regions and did not test the same FNIRT protocol as that used in this study. Here we build on these results in a larger study group and across a wider range of WM fasciculi using a more appropriate metric of assessment.

The registration methods explored here both use a template that is averaged from the study cohort, which improves image alignment accuracy (Van Hecke et al., 2011). The scalar-based registration evaluated here was improved for neonatal populations by introducing an extra linear registration step and registering all the subjects to the mean FA map created after first registering to the most representative subject. This improved alignment over standard TBSS registration, which previously failed for some subjects (Bassi et al., 2008). TSA's algorithm uses an iteratively refined template averaged from all subjects’ tensor images. Keihaninejad et al. (2012) showed that alignment was improved when registering to an iteratively refined template over registering to the mean FA.

Our analysis presents a novel approach to assessing registration accuracy in the neonatal population. Previous studies have focused on using image similarity measures and tissue label overlap scores to assess registration performance. However it has been shown that these are not reliable criteria for establishing registration accuracy and that only local labeled ROIs are able to appropriately distinguish registration performance (Rohlfing, 2012).

We report similar Dice score results to those seen in previous registration comparison studies. Klein et al. (Klein et al., 2009) reported similar values in overlap measures in an evaluation of 14 different registration methods with adult subjects. They obtained slightly higher measure of overlap only when looking at larger regions which cover a greater extent of the brain. It should be noted that by looking at smaller local ROIs even small disagreements in overlap can lower the Dice score. Moreover we are looking at a relatively large age range which may explain the high variability observed in native space tracts.

#### Data projection

We present the first analysis of the concordance between template space data projected onto WM skeletons derived from TSA and TBSS and native space data. The closer agreement between FA values derived from native space tractography and FA values projected by TSA demonstrates that the TSA skeleton model is able to represent more accurately individual subjects’ tracts than TBSS. The discrepancy between TSA-projected, TBSS-projected and native space-derived FA values is most likely due to (i) dimensionality reduction in TBSS and TSA from volumetric tracts to voxel-wise skeleton and surface skeleton, respectively; (ii) the projection of the maximum FA value; and (iii) misregistrations between the template and subject. The closer agreement between the values projected by TSA and native space tractography than those projected by TBSS may be due to better-defined stopping criteria for the data projection search in TSA. The search for the maximum FA value in TBSS can cross over into neighbouring tracts (Bach et al., 2014) and the different maturation rates for different tracts during early development (Nossin-Manor et al., 2015) may compound this. Moreover, the data projection in TBSS aligns voxels with the same FA rather than voxels from the same anatomical structure (Zalesky, 2011).

### Correlation of DT-derived measures and post-menstrual age at scan using TSA

Our results show a maturation-dependent increase in FA and decrease in diffusivity that concurs with previous studies in preterm infants (Bonifacio et al., 2010, Nossin-Manor et al., 2015, Partridge et al., 2004). Age-related changes in the CC were localised to the splenium and genu for FA and RD, and in the cortical projections for MD and very little correlation with AD. (Rose et al., 2014) found no correlations between DTI metrics in the CC and PMA in preterm infants scanned near term equivalent age (TEA) using a ROI method averaging over the whole tract. Considering that the effect observed here is not widespread it is possible that such approaches would fail to detect smaller, localised changes previously observed in the genu (Gilmore et al., 2007) and splenium (de Bruine et al., 2011). Using TSA, we can assess the CC in its entirety without obscuring regional changes or needing to segment the tract into its constituent parts. The CST showed age-dependent changes in DTI metrics in the central semiovale and posterior limb of the internal capsule similar to previous studies (Aeby et al., 2009, Partridge et al., 2004).

Association fibres such as the SLF, ILF and IFOF can be difficult to identify at birth in term infants as they mature more slowly (Hermoye et al., 2006). We were able to delineate these tracts using TSA, apart from the temporal projections of the right SLF, demonstrating that TSA can be applied successfully to neonatal populations. We identified a maturation pattern previously observed in preterm infants (Nossin-Manor et al., 2015) and term infants (Oishi et al., 2011).

FA values in the UNC demonstrated a strong positive correlation with PMA at scan and we observed a negative correlation between MD and RD in the frontal lobe projections with increasing PMA. The UNC has been shown to be affected by preterm birth (Constable et al., 2008) and similar pattern of increased FA and decreased diffusivity with increasing PMA has been detected in term infants (Dubois et al., 2006) however it has not been studied extensively in preterm infants in the perinatal period. Here we demonstrate that TSA is appropriate for such analysis.

Our results show limited correlation between AD and PMA. Previous studies have reported differing results for changes in AD associated with age at scan. Studies with similarly-sized cohorts found age-related changes only in the anterior limb of the internal capsule and the thalamus (Rose et al., 2014, Seo et al., 2013) while larger studies (Ball et al., 2010, Kersbergen et al., 2014) report wider changes across the WM. The absence of age-related changes in AD indicate that changes in FA are driven by reductions in RD, in line with previous studies (Adams et al., 2010). These changes are likely to reflect myelination and premyelination events such as increases in axon diameter and decreased membrane permeability, oligodendrocyte proliferation and maturation, resulting in more coherent axonal organization and overall reduction in free water (Beaulieu, 2002, Wimberger et al., 1995).

We found no correlation between GA at birth or post-natal day at scan and DTI metrics. Prematurity at birth has been shown to affect DTI metrics in larger cohorts (Ball et al., 2010), while a similarly-sized study (n=45) (Rose et al., 2014) found no correlations between DTI metrics and GA, apart from changes in diffusivity in the extreme capsule, anterior limb of the internal capsule, retrolenticular part of the internal capsule and the putamen.

### Advantages and limitations of TSA

TSA has several advantages as an analysis tool. It is automated; its surface representations of tracts approximate closely native space diffusion data and TSA offers improved alignment between WM tracts.

A major benefit of TSA is that it is anatomically specific, like other tracts-of-interest methods. Tract-of-interest approaches using tractography have been previously used to study development, averaging DTI values over the whole tract (Berman et al., 2005, Braga et al., 2015, Miller et al., 2002). The axonal configuration of a tract may vary along its length resulting in different diffusion properties at different locations. WM development follows an asynchronous spatio-temporal pattern (Oishi et al., 2011) and differences in FA between subjects born preterm and controls and have been shown to be non-uniform along WM tracts (Groeschel et al., 2014, Travis et al., 2015, Yeatman et al., 2012). In this respect, TSA offers the benefits of anatomically specific analysis with the ability to highlight regions of statistical significance at locations along a tract, and is less likely to obscure localised differences. The downside to using TSA is that the investigator must assume a priori where they expect to observe a change of interest. When no such information is available exploratory, whole-brain analyses like TBSS may be more appropriate.

A possible application of TSA could be to study subjects where tractography is unsuccessful due to brain injury. As the tract skeletons are defined in the template it would be possible to project such subjects’ diffusion data onto the skeleton, as long as the registration was successful.

A limitation of TSA, as assessed here, is the geometry it is able to describe. The choice made here, the FACT algorithm for tractography, is motivated by its suitability to the diffusion data at hand (32 gradient directions and a b-value of 750 s/mm^2^).

While most of the TSA pipeline is automated, we have chosen to identify tracts in the study-specific template with manually-drawn ROIs to maximize the accuracy of the tract delineation. This step may be further automated with atlas-based techniques which have proven to be successful in recent studies (Akazawa et al., 2016, Oishi et al., 2011). Such an approach may be beneficial for large studies such as the Developing Human Connectome Project (Hughes et al., 2016) where multiple study-specific templates may be required.

We have also omitted tracts that have a tubular structure, such as the fornix and cingulum. This is because TSA is ill-suited to such structures. The tract skeleton is determined by thinning the tract-boundary down to a medial surface. This strategy is poorly defined when there are multiple directions in which it possible to thin, as would be the case for cylindrical structures. For such tracts, it would be more appropriate to use methods such as those developed by (Corouge et al., 2006, O'Donnell et al., 2009, Yeatman et al., 2012).

As part of our evaluation of TSA we have analysed only DTI-derived measures, however the framework allows data from other diffusion models, such as NODDI (Tariq et al., 2016, Zhang et al., 2012) or g-ratio mapping (Stikov et al., 2015), to be projected onto the skeletons.

## Conclusions

We evaluate the performance of TSA against native space tractography, which serves as the gold standard, using TBSS as a benchmark, for the preterm population. This work demonstrates that TSA is a suitable method for infant studies using dMRI when particular tracts are to be targeted. The framework allows numerous WM tracts to be analysed and, by design, can easily be applied to large cohort studies. Here we have demonstrated the effects of age at scan on DTI metrics in nine tracts showing that TSA is indeed sensitive to the developmental changes.
